# Adjunctive Probiotic *Lactobacillus rhamnosus* Probio-M9 Administration Enhances the Effect of Anti-PD-1 Antitumor Therapy *via* Restoring Antibiotic-Disrupted Gut Microbiota

**DOI:** 10.3389/fimmu.2021.772532

**Published:** 2021-12-14

**Authors:** Guangqi Gao, Teng Ma, Tao Zhang, Hao Jin, Yalin Li, Lai-Yu Kwok, Heping Zhang, Zhihong Sun

**Affiliations:** ^1^ Inner Mongolia Key Laboratory of Dairy Biotechnology and Engineering, Key Laboratory of Dairy Biotechnology and Engineering, Ministry of Education, Inner Mongolia Agricultural University, Hohhot, China; ^2^ Key Laboratory of Dairy Products Processing, Ministry of Agriculture and Rural Affairs, Inner Mongolia Agricultural University, Hohhot, China

**Keywords:** gut microbiota, anti-PD-1, antibiotic, probiotic, *Lactobacillus rhamnosus* Probio-M9, synergistic tumor therapeutics

## Abstract

Emerging evidence supports that the efficacy of immune checkpoint blockade (ICB) therapy is associated with the host’s gut microbiota, as prior antibiotic intake often leads to poor outcome and low responsiveness toward ICB treatment. Therefore, we hypothesized that the efficacy of ICB therapy like anti-programmed cell death protein-1 (PD-1) treatment required an intact host gut microbiota, and it was established that probiotics could enhance the recovery of gut microbiota disruption by external stimuli. Thus, the present study aimed to evaluate the effect of the probiotics, *Lactobacillus rhamnosus* Probio-M9, on recovering antibiotic-disrupted gut microbiota and its impact on the outcome of ICB therapy in tumor-bearing mice. We first disrupted the mouse microbiota by antibiotics and then remediated the gut microbiota by probiotics or naturally. Tumor transplantation was then performed, followed by anti-PD-1-based antitumor therapy. Changes in the fecal metagenomes and the tumor suppression effect were monitored during different stages of the experiment. Our results showed that Probio-M9 synergized with ICB therapy, significantly improving tumor inhibition compared with groups not receiving the probiotic treatment (*P* < 0.05 at most time points). The synergistic effect was accompanied by effective restoration of antibiotic-disrupted fecal microbiome that was characterized by a drastically reduced Shannon diversity value and shifted composition of dominating taxa. Moreover, probiotic administration significantly increased the relative abundance of beneficial bacteria (e.g., *Bifidobacterium pseudolongum*, *Parabacteroides distasonis*, and some *Bacteroides* species; 0.0001 < *P* < 0.05). The gut microbiome changes were accompanied by mild reshaping of the functional metagenomes characterized by enrichment in sugar degradation and vitamin and amino acid synthesis pathways. Collectively, this study supported that probiotic administration could enhance the efficacy and responsiveness of anti-PD-1-based immunotherapy, and Probio-M9 could be a potential candidate of microbe-based synergistic tumor therapeutics. The preclinical data obtained here would support the design of future human clinical trials for further consolidating the current findings and for safety assessment of probiotic adjunctive treatment in ICB therapy.

## Introduction

Immunotherapy is a relatively new method for scientific research and clinical treatment to a variety of malignant tumors (1). It is realized by immune checkpoint blockade (ICB), which activates T lymphocyte-mediated immune response to improve tumor immunosurveillance (2, 3). Immune checkpoint inhibitors (ICIs) in the tumor microenvironment suppress the immune escape of tumor cells by antagonizing the negative regulators on T cells (4). This class of molecules includes cytotoxic T lymphocyte-associated antigen-4 (CTLA-4), programmed cell death protein-1 (PD-1), and programmed death-ligand 1 (PD-L1), which are the three major targets for ICB therapies used in current clinical practice (5). The key regulator pair of immune checkpoint, PD-1 and its ligand, PD-L1, maintains the immune balance of organisms by regulating T-cell and B-cell activities (6). Under normal circumstances, antigen-presenting cells (APCs) activate T cells through costimulatory signals and release interferon gamma (IFN-γ) to induce immune cells to express PD-L1. When PD-L1 binds to PD-1 on the surface of T cells, it inhibits the activities of Zeta-chain associated protein 70 (ZAP70) and phosphoinositide 3-kinase (PI3K) by recruiting Src homology region 2 (SH-2) domain-containing phosphatase 1(SHP1) and SH-2 domain-containing phosphatase 2 (SHP2) phosphatases and activating Phosphatase and tensin homologue deleted on chromosome ten (PTEN) phosphatases, blocks the costimulation between T cells and APC, and leads to a reduction in T-cell function (6, 7). However, some cancer cells can cause T-cell dysfunction through the expression of PD-L1 molecules, thus avoiding the monitoring and killing of tumor cells and promoting the growth of tumor (7). Therefore, one strategy of tumor immunotherapy relies on using anti-PD-1 or anti-PD-L1 monoclonal antibodies to antagonize the targeted molecules of corresponding immune checkpoint, thus restoring T-cell cytotoxic activity against tumor cells.

The ICB therapy has been successfully used in clinical treatment of solid tumors, such as melanoma, renal cell carcinoma, non-small cell lung cancer, and DNA mismatch repair-deficient colorectal cancer. The overall and objective response rates of some patients have improved significantly after immunotherapy (8). However, only few patients benefitted from ICIs (9) partly due to individual differences between patients (7, 10). Emerging evidence supports that the efficacy of ICB therapy correlates with signatures of the host gut microbiota (11, 12). The use of antibiotics impairs the efficacy of cancer therapies in both mice and patients (13, 14), which is possibly due to gut dysbiosis and drastic reduction in the beneficial microbial subpopulation. Thus, target modulation of the gut microbiome by means of products and management approaches, such as probiotics, prebiotics, dietary intervention, and fecal microbiota transplantation, could be potential strategies for promoting the clinical efficacy of ICB treatment (15). For instance, *Bifidobacterium* species and *Akkermansia muciniphila* have been shown to enhance the host immune responses toward ICB therapy by promoting antigen presentation of dendritic cells (13, 16–19). Besides, recent studies found that *Lactobacillus rhamnosus* GG could improve the antitumor immunity of mice (20, 21). The findings of these investigations support that the administration of exogenous probiotics could be a promising way to improve the therapeutic efficacy of ICB. Notably, the synergistic effect of probiotics with ICIs in tumor suppression seemed strain-specific (22); it is therefore necessary to evaluate the therapeutic efficacy of individual candidate probiotic strains in ICB treatment.

Our previous work isolated a probiotic strain from human colostrum, *L. rhamnosus* Probio-M9, which exhibited inhibitory effects on colitis-associated carcinogenesis possibly *via* restoring the gut microbiota (23, 24). We hypothesized that the efficacy of anti-PD-1-based antitumor therapy required an intact host gut microbiota, and it was established that probiotics could modulate and enhance the recovery of gut microbiota disruption by external stimuli. Thus, the present study aimed to evaluate the effect of this strain on recovering antibiotic-disrupted gut microbiota and its impact on the efficacy of ICB therapy in a heterotopic *in vivo* model of colorectal cancer.

## Materials and Methods

### Animals, Cells, and Probiotics

This study was approved by the Special Committee on Scientific Research and Academic Ethics of Inner Mongolia Agricultural University (No. 2020-049). Specific pathogen-free (SPF) BALB/c mice were purchased from Beijing Vital River Laboratory Animal Technology Co., Ltd. (Beijing, China). Six- to 8-week-old mice were raised and housed under SPF conditions and received sterilized feed and water. The mouse colorectal cancer cell line, CT26.WT (ATCC^®^ CRL-2638), was cultured in RPMI-1640 medium (Gibco, USA) containing 10% fetal bovine serum (Gibco, USA) in a humidified incubator (Thermo, USA) at 37°C, 5% CO_2_. The probiotic strain, Probio-M9, was provided by the Key Laboratory of Dairy Biotechnology and Engineering, Ministry of Education, Inner Mongolia Agricultural University, China.

### Experimental Design

**Figure 1:**
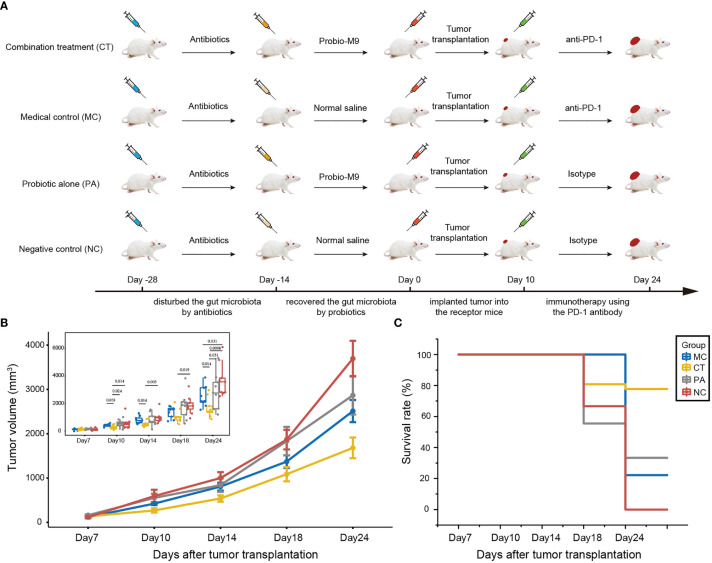
Effect of host microbiota modulation on antitumor therapy by anti-programmed cell death protein-1 (PD-1) antibody. **(A)** Schematic diagram showing the experimental design. The experiment was performed in four sequential stages. Antibiotics was first applied to disrupt the host gut microbiota, followed by its reconstruction by the probiotics, Probio-M9 (control groups received saline), prior to tumor transplantation. Finally, the tumor suppression effect of anti-PD-1 antibody (control groups received rat isotype antibody) was applied to hosts of different backgrounds of modulated microbiota. **(B)** CT26 tumor growth kinetics and **(C)** survival rate of the four groups of mice. Significant differences were evaluated by Wilcoxon test; only significant differences (*P* < 0.05) are shown. Data were expressed as means ± SEM (n = 9).

The design of the mouse experiment is shown in **Figure 1A**. Mice were randomized into four groups: medical control (MC), combination treatment (CT), probiotic alone (PA), and negative control (NC) (n = 9 per group). The experiment was carried out in four sequential stages: antibiotic treatment, probiotic intervention, tumor growth, and anti-PD-1 immunotherapy. For antibiotic treatment, an antibiotic cocktail was prepared in autoclaved drinking water for 2 weeks with ampicillin (1 g/L), metronidazole (1 g/L), neomycin (1 g/L), and vancomycin (0.5 g/L) (25). Probiotics were administered to mice from CT and PA groups by daily oral gavage with 5 × 10^9^ CFU Probio-M9 for 2 weeks prior to tumor inoculation, and the two non-probiotic recipient groups were given an equal amount of normal saline. Tumor transplantation was conducted at day 0, and all mice were subcutaneously injected with 5 × 10^5^ CT26 cells. For ICB therapy, mice from MC and CT groups were intraperitoneally injected with 100 μg anti-mouse PD-1 monoclonal antibody (Clone RMP1-14, BioXCell, USA), while mice from PA and NC groups were intraperitoneally injected with 100 μg rat immunoglobulin (Ig)G2a (Clone 2A3, BioXCell, USA) as isotype control (17, 26). The injections were administered every 3 or 4 days from day 10, and the mice were sacrificed at day 24. Tumor size was measured at days 7, 10, 14, 18, and 24 after tumor cell injection, and tumor volume was determined as length × width^2^ × 0.5 (16). Survival rate was defined as the percentage of mice with tumor volume of less than 2,000 mm^3^ in each group.

### Fecal Sample Collection and Metagenomic Shotgun Sequencing

Mouse feces were sampled at six different time points, which were days -28, -14, 0, 10, 18, and 24 (**Figure 1A**), representing the gut microbiota at baseline, after antibiotic treatment, probiotic treatment, tumor transplantation, half course of medical intervention, and complete course of treatment, respectively. To collect fecal samples, the mouse was fixed by the animal handler, and then its abdomen was gently massaged to facilitate excretion. Fecal pellets were directly collected in 1.5 ml Eppendorf tubes and stored at -80°C immediately. Prior to metagenomic sequencing, the fecal samples were thawed, and total genomic DNA was extracted using the QIAamp Fast DNA Stool Mini Kit (Qiagen, Hilden, Germany). Libraries were constructed using NEBNext^®^ Ultra™ DNA Library Prep Kit for Illumina (NEB, USA) following the manufacturer’s recommendations to generate DNA fragments of ~300 bp length. Paired-end reads were generated by sequencing (Illumina NovaSeq 6000 platform) 150 bp length in both forward and reverse directions. The reads were quality-controlled by trimming the low-quality reads using the KneadData pipeline (http://huttenhower.sph.harvard.edu/kneaddata; v0.7.5) and were subsequently aligned to the mouse genome to remove host DNA sequences using Bowtie2 (v2.3.5.1) (27) under default parameters. A total of 1,851.96 Gb raw data (mean = 11.43 Gb data per sample) were generated and were filtered to produce 881.88 Gb high-quality clean data.

### Identification of Microbial Species and Metabolic Pathways

The shotgun reads were assembled into contigs using MEGAHIT with the default parameter. Kraken2 (28) was applied for annotation of metagenomic species, while the functional metagenome and corresponding metabolic pathways were annotated by HUMAnN2 pipeline (29) based on the UniRef90 database (https://www.uniprot.org/help/uniref).

### Statistical Analyses

All statistical analyses were performed using R software (v.4.0.2). Principal coordinates analysis (PCoA) was performed and visualized using R packages (vegan and ggpubr), while the adonis *P* value was generated based on 999 permutations. Kruskal–Wallis test, Wilcoxon test, and t-test were used to evaluate differences in various variables between groups; *P* values were corrected for multiple testing using the Benjamini–Hochberg procedure. Pathway enrichment was calculated by STAMP software (v.2.1.3). All graphical presentations were generated under the R and Adobe Illustrator environment.

## Results

### Probio-M9 Administration Improved the Efficacy of Anti-PD-1-Based Immunotherapy

This work performed a four-stage experiment to first disrupt the mouse microbiota by antibiotics, followed by remediation of the gut microbiota by probiotics. The tumor suppression effect of anti-PD-1-based antitumor therapy in mice having different backgrounds of microbiota modulation was compared (**Figure 1A**).

At day 24, the mean tumor volume of CT group (both anti-PD-1 antibody and probiotics; mean tumor size ± SEM = 1,681.02 ± 77.86 mm^3^) was the smallest, followed by MC group (received anti-PD-1 antibody but not probiotics; mean tumor size ± SEM = 2,511.05 ± 83.64 mm^3^), PA group (received probiotics but not anti-PD-1 antibody; mean tumor size ± SEM = 2,867.67 ± 144.60 mm^3^), and NC group (did not receive probiotics nor anti-PD-1 antibody; mean tumor size ± SEM = 3,695.74 ± 134.39 mm^3^) (**Figure 1B**). Notably, the anti-PD-1 treatment significantly reduced the tumor volume compared with other groups (*P* < 0.05 at day 24), suggesting that the anti-PD-1 treatment was effective in tumor suppression with or without probiotic supplementation. No significant difference was seen in the tumor volume between PA and NC groups at any monitored time point, suggesting that administration of Probio-M9 alone had no significant effect on tumor suppression. However, interestingly, probiotic supplementation synergized the antitumor effect of anti-PD-1 treatment (mean tumor size ± SEM of CT and MC groups = 538.63 ± 24.03 and 807.29 ± 24.03 mm^3^ at day 14, *P* = 0.014; 1,087.22 ± 53.67 and 1,371.28 ± 47.01 mm^3^ at day 18, *P* = 0.258; 1,681.02 ± 77.86 and 2,511.05 ± 83.64 mm^3^ at day 24, *P* = 0.014), indicating that the synergistic effect was seen as early as 4 days after the first anti-PD-1 antibody injection (**Figure 1B**).

Consistent with the efficacy in suppression of tumor volume, CT group had the highest animal survival rate (77.8% vs. 33.3%, 22.2%, and 0% in PA, MC, and NC groups, respectively; **Figure 1C**). Notably, the survival rate of MC group (anti-PD-1 treatment alone) was only slightly higher than that of NC group (non-treatment control), suggesting that the synergistic tumor suppression effect of probiotics was required for the survival of some of the tumor-bearing mice (**Figure 1C**).

### Metagenomics Revealed Changes in Gut Microbiota by Antibiotic and Probiotic Treatment

To assess the dynamic changes of gut microbiota under different treatments, metagenomic sequencing was performed on 162 mouse fecal samples [six sampling time points × three groups (CT, MC, and NC) × nine mice per group]. It should be noted that fecal samples collected from PA group were not included in the metagenomic sequencing, as there was no significant efficacy in tumor suppression compared with NC group. The information on samples that underwent metagenomic sequencing and the amount of generated data are tabulated (**Table 1**).

**Table 1:** Amount of generated metagenomic sequencing data.

Group (n = 9)	Sampling time	Group label	Raw data (Gb)	Clean data (Gb)
MC	Day -28	MC1	11.95	5.96
CT	Day -28	CT1	11.59	5.41
NC	Day -28	NC1	10.90	5.61
MC	Day -14	MC2	11.10	5.31
CT	Day -14	CT2	11.26	5.41
NC	Day -14	NC2	11.28	5.54
MC	Day 0	MC3	11.10	5.66
CT	Day 0	CT3	11.00	5.19
NC	Day 0	NC3	11.14	5.62
MC	Day 10	MC4	10.98	5.08
CT	Day 10	CT4	12.25	5.88
NC	Day 10	NC4	11.34	5.47
MC	Day 18	MC5	12.47	5.32
CT	Day 18	CT5	11.75	5.85
NC	Day 18	NC5	11.44	5.63
MC	Day 24	MC6	10.55	4.67
CT	Day 24	CT6	11.90	4.83
NC	Day 24	NC6	12.48	5.55

MC, medical control; CT, combination treatment; NC, negative control.

**Figure 2:**
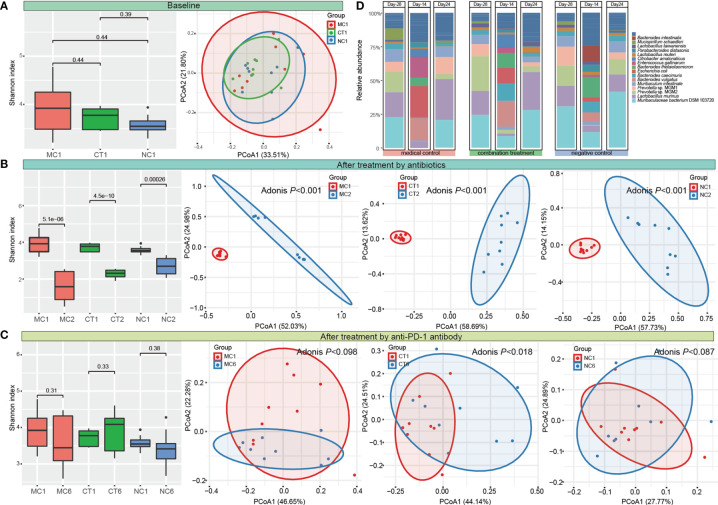
Dynamic changes of diversity, structure, and composition of gut microbiota. Shannon diversity index and principal coordinates analysis (PCoA, Bray–Curtis) of gut microbiota **(A)** at the baseline, **(B)** after antibiotic treatment, **(C)** at the end of the intervention. MC, medical control; CT, combination treatment; NC, negative control. The number after the group label represents the corresponding sampling time point (1: Day -28, 2: Day -14, and 6: Day 24). **(D)** Fecal microbiota composition of MC, CT, and NC groups at Day -28, Day -24, and Day 24 (representing the time points right before antibiotic and probiotic treatments and at the end of anti-programmed cell death protein-1 (PD-1) target therapy, respectively). T-test and Adonis test were used to evaluate the differences between the groups.

Microbial diversity and composition were analyzed to evaluate the changes of gut microbiota induced by antibiotic and probiotic administration. No significant difference was found in the Shannon diversity, and the PCoA score plot did not show obvious group-based clustering pattern (**Figure 2A**), indicating that there was no apparent difference in the overall microbiota diversity and structural difference between groups before any treatment started. After 2 weeks of antibiotic treatment, the Shannon index decreased (*P* < 0.001), while the Bray–Curtis distance increased significantly (Adonis test, *P* < 0.001) compared with the baseline, suggesting that the diversity and structure of gut microbiota were disrupted by antibiotics (**Figure 2B**). Then, each group of mice was further treated according to the experimental design by probiotic intervention or natural recovery of the antibiotic-disrupted gut microbiota. At day 24, the level of Shannon diversity index in all groups returned to the baseline level, while the gut microbiota structure of only CT group (received combination treatment) but not MC and NC groups (neither of the groups received probiotics) was significantly different compared with baseline (Adonis test, *P* < 0.018; **Figure 2C** and **Figure S1**), suggesting that the gut microbiota could recover naturally without external intervention, and Probio-M9 intervention modulated the gut microbiota differently from that *via* natural recovery.

Meanwhile, the fecal microbial composition was also changed after administering antibiotics and probiotics. The main components of gut bacteria were *Muribaculaceae bacterium* DSM 103720 (22.86%–30.78%), *Lactobacillus murinus* (15.25%–18.42%), and *Prevotella* (14.48%–34.59%) initially. Antibiotic treatment greatly eliminated the originally dominating taxa and led to the predominance of *Escherichia coli*, *Enterococcus gallinarum*, and *Bacteroides* species in bacterial community (the sum proportion of these three taxa accounted for >51.87% in each group). At day 24, the relative abundance of most taxa returned to their original levels in all groups (**Figure 2D** and **Table S1**).

### Effect of Antibiotics, Probio-M9, and Anti-PD-1 Treatments on the Key Responsive Bacterial Species

To further analyze the effect of antibiotics or probiotics on the composition of gut microbiota in mice, we then identified significant differentially abundant species that were responsive to these treatments. Overall, there were 13 significant differentially abundant species that were responsive to the antibiotic treatment, among which the relative abundance of *A. muciniphila*, *Bacteroides vulgatus*, *E. gallinarum*, and *Escherichia coli* significantly increased, while other species (*Anaerotruncus* sp. G32012, *Bacteroides faecichinchillae*, *Bacteroides uniformis*, *Dorea* sp. 52, *Firmicutes bacterium* ASF500, *M. bacterium* DSM 103720, *Muribaculum intestinale*, *Prevotella* sp. MGM1, and *Prevotella* sp. MGM2) significantly decreased (*P* < 0.05; **Figures S2A, B**).

After antibiotic treatment, the gut microbiota was recovered either naturally or by probiotic intervention for 2 weeks. The relative abundance of six species (*B.vulgatus*, *Citrobacter amalonaticus*, *Clostridium* sp. ASF502, *E. coli*, *M. bacterium* DSM 103720, and *Prevotella* sp. MGM2) in CT group (Probio-M9 treated) was significantly higher than those in the other two groups (MC, NC; *P* < 0.05), and the proportion of *Clostridium* sp. ASF502, *M. bacterium* DSM 103720, and *Prevotella* sp. MGM2 increased from day -14 to day 0 only after Probio-M9 gavage (*P* < 0.05). Such trends were not observed in the other two groups without Probio-M9 (**Figures S2C, D**) . In addition, we noted that Probio-M9 treatment increased the relative abundance of *Bifidobacterium pseudolongum* by 136-fold, while it decreased *A. muciniphila* by 12-fold, although their changes were statistically non-significant (*P* = 0.078 for *B. pseudolongum* and *P* = 0.068 for *A. muciniphila*; **Figure S2D**). These results suggested that there was great individual variation in gut microbiota responses toward Probio-M9 treatment, but some species, e.g., *A. muciniphila* and *B. pseudolongum*, were responsive toward the treatment in most mice.

**Figure 3:**
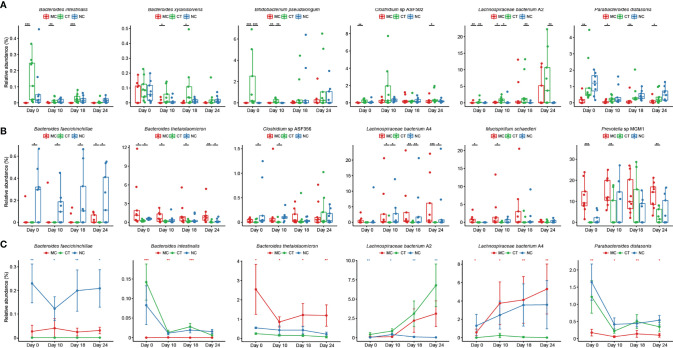
Probio-M9-specific modulation of key microbial species. Species significantly increased **(A)** and decreased **(B)** after Probio-M9 supplementation. **(C)** Species exhibited significant changes after the course of intervention. Groups: medical control (MC), combination treatment (CT), negative control (NC). Error bars represent SEM. *P < 0.05; **P < 0.01; ***P < 0.001.

Next, the effect of Probio-M9 intervention on host gut microbiota during anti-PD-1 treatment was investigated. We identified species showing significant changes due to probiotics rather than antibiotics, i.e., species showing no significant changes after antibiotic treatment but exhibiting significant changes in relative abundance from day 0 to day 24. Significantly more *Bacteroides intestinali*s, *Bacteroides xylanisolvens*, *B. pseudolongum*, *Clostridium* sp. ASF502, *Lachnospiraceae bacterium* A2, and *Parabacteroides distasonis* were found in CT group than MC or NC group (**Figure 3A**; 0.0001 < *P* < 0.05), and opposite trends were observed in *Bacteroides faecichinchillae*, *Bacteroides thetaiotaomicron*, *Prevotella* sp. MGM1, *Mucispirillum schaedleri*, *Clostridium* sp. ASF356, and *Lachnospiraceae bacterium* A4 (**Figure 3B**; 0.001 < *P* < 0.05). The relative abundance of *B. faecichinchillae*, *B. intestinalis*, *B. thetaiotaomicron*, *L. bacterium* A2, *L. bacterium* A4, and *P. distasonis* fluctuated significantly in the process of tumor growth and treatment (**Figure 3C**).

### Effect of Antibiotics, Probio-M9, and Anti-PD-1 Treatment on the Functional Potential of the Gut Microbiome

Since antibiotics and probiotics influenced the structure and composition of the mouse gut microbiota, changes in the functional fecal metagenome were anticipated. The functional genes in the fecal metagenome were analyzed by HUMAnN2 pipeline and returned 485 pathways (**Table S2**). Antibiotic administration altered the function of the gut microbiome drastically, as the gene abundance of 272 pathways changed significantly across the three groups (*P* < 0.05; **Table S3**). Nine significant differentially abundant pathways were identified between the probiotic recipients (CT group) and non-probiotic recipients (MC and NC groups), which was likely due to the exogenous Probio-M9 supplementation (CT group *P* < 0.05, MC and NC groups *P* > 0.05; **Table S4**).

**Figure 4:**
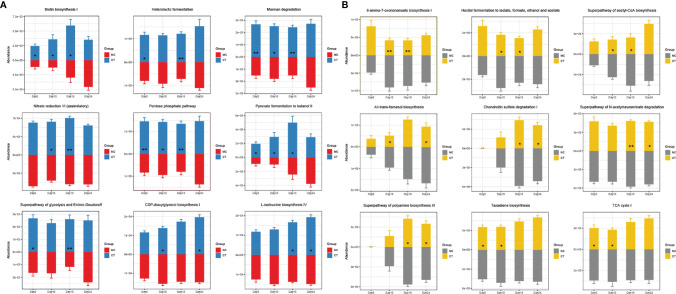
Effects of Probio-M9 administration on gut metagenomic potential. Gene abundance of pathways significantly increased **(A)** and decreased **(B)** by Probio-M9 supplementation. Groups: medical control (MC); combination treatment (CT). *P < 0.05; **P < 0.01.

Then, during the stage of tumor implantation and anti-PD-1 treatment, the gene abundance of several pathways, including biotin biosynthesis I, heterolactic fermentation, mannan degradation, nitrate reduction VI (assimilatory), pentose phosphate pathway, pyruvate fermentation to butanol II, superpathway of glycolysis and Entner–Doudoroff, CDP-diacylglycerol biosynthesis I, and L-isoleucine biosynthesis IV, increased in CT group, while some other pathways, such as 8-amino-7-oxononanoate biosynthesis I, hexitol fermentation to lactate, formate, ethanol, and acetate, superpathway of acetyl-coA biosynthesis, all-trans-farnesol biosynthesis, chondroitin sulfate degradation I, superpathway of N-acetylneuraminate degradation, superpathway of polyamine biosynthesis III, taxadiene biosynthesis and TCA cycle I, were less abundant (**Figure 4**). Such probiotic-driven changes in the functional gut metagenome were maintained during the anti-PD-1 immunotherapy, and they could be the reason for the synergistic tumor suppressive effect of Probio-M9.

## Discussion

The efficacy of ICB therapy is affected by the host gut microbiota (16–18, 30), and the non-responsive rate tends to increase in patients taking antibiotics before receiving immunotherapy (31). Antibiotics affect the host’s physiological response by drastically shifting the gut microbiota structure and altering the composition of gut commensals, which in turn impact the host’s immunity (32). Probiotics are live bacteria that confer beneficial effects on the host, and one of the beneficial mechanisms is *via* regulating the host gut microbiota (33), further modulating the host immune responses and subsequently enhancing the responsiveness toward immunotherapy. This study evaluated the effect of the probiotic, *L. rhamnosus* Probio-M9, on anti-PD-1-based ICB treatment using a heterotopic *in vivo* model of colorectal cancer constructed in mice with antibiotic-disrupted gut microbiota. It is interesting to note that taking probiotics after administration of antibiotics enhanced the tumor growth inhibitory effect in subsequent ICB treatment; such tumor inhibitory effect was significantly stronger than that in non-probiotic-treated mice (*P* < 0.05), which was consistent with the observations of a previous study (24), showing that Probio-M9 could promote the therapeutic efficacy in both orthotopic and ectopic colon cancer.

This work collected mouse fecal samples at different stages of the animal experiment, and the samples were subjected to metagenomic sequencing to monitor the dynamic changes of gut microbiome during/after antibiotic, probiotic, and anti-PD-1 treatments. The use of antibiotics not only decreased the diversity of microbiota but also changed the composition of the fecal microbiome. Antibiotic application selected the spectrum of antibiotic-resistant bacteria, particularly *A. muciniphila*, and meanwhile increased the proportion of other potentially harmful ones, such as *E. coli* and *E. gallinarum*. These species are known to be associated with tumorigenesis and even related to the non-responsiveness of patients in ICB treatment (30, 34). Probio-M9 could effectively restore the gut microbiota diversity and structure of mice previously treated by antibiotics. The restoration of the disrupted gut microbiota was conducive to the subsequent anti-PD-1 immunotherapy.

**Figure 5:**
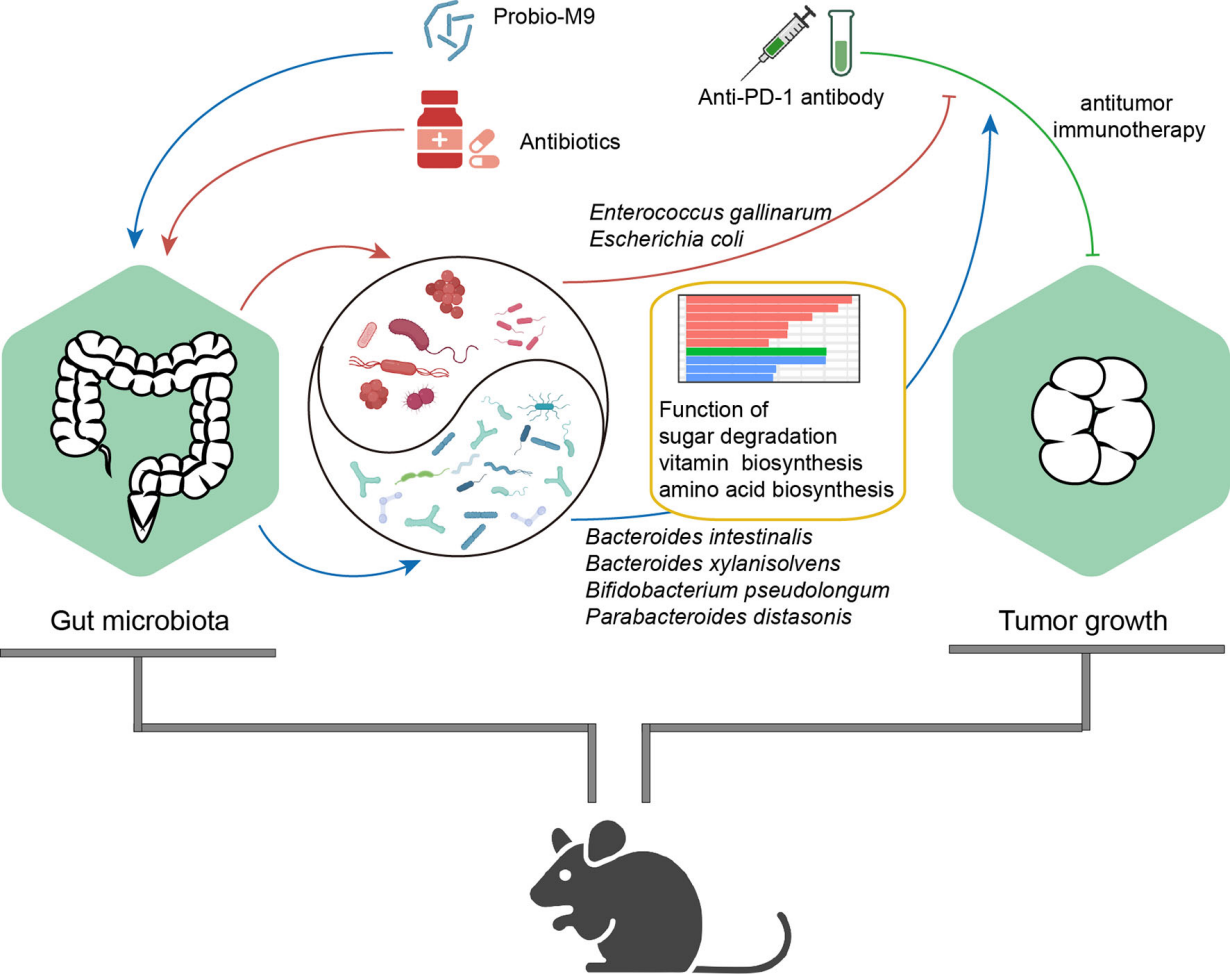
Proposed mechanism of synergistic antitumor effect of the probiotics, Probio-M9, in anti-programmed cell death protein-1 (PD-1) treatment *via* improving antibiotic-disrupted gut microbiota. Red lines and blue lines indicate the effects of antibiotics or Probio-M9 on the gut microbiota previously modulated by antibiotics or Probio-M9, respectively. Green line indicates the antitumor activity of anti-PD-1 therapy. Arrows and blunt ends indicate promoting and inhibiting effects, respectively.

Our data showed that one of the most predominant genera in the mouse gut, *Bacteroides*, was significantly affected by antibiotics and probiotics. Probio-M9 increased the relative abundance of *B. intestinalis* and *B. xylanisolvens*, which were considered to be beneficial to host health (35). The species *B. intestinalis* could enhance host immunity by producing metabolites or inducing transcription of interleukin (IL)-1β (36, 37), and *B. xylanisolvens* correlated positively with cancer treatment outcomes (38). In another study, the relative abundance of *B. thetaiotaomicron*, a species that was reported to be enriched in patients who were non-responsive to ICB treatment (30), decreased by Probio-M9 administration. Therefore, it was likely that Probio-M9 had distinct regulatory effects on different colonic *Bacteroides* species. Besides, some bacterial strains from the *Clostridium* genus and *Lachnospiraceae* family were also influenced by Probio-M9 specifically. Previous studies concluded that *Lachnospiraceae* were related to immune cell development and anti-inflammatory function, and they were enriched in patients responsive to ICB treatment (39–41); thus, it would be of interest to further investigate their exact role in immunomodulation, particular identifying strains responsible for regulating the host’s immune function. Notably, the abundance of *P. distasonis* and *B. pseudolongum* increased significantly after Probio-M9 supplementation. The species *P. distasonis* was found to be associated with effectiveness in antitumor immunotherapy, and it was considered as a predictive indicator of response to combined ICB treatment (37), while *B. pseudolongum* could enhance immunotherapy response through metabolite production (18). The results of this study are largely consistent with published reports, suggesting that administering probiotics (Probio-M9 in this case) promoted the antitumor immune response in anti-PD-1-based therapy by enhancing the beneficial bacteria while suppressing the harmful ones in antibiotic-treated tumor-bearing mice (**Figure 5**).

As the gut microbiota of the mice was shaped by the actions of antibiotics and probiotics, the functional metagenome of the mice that underwent anti-PD-1 treatment was also modulated. The functional metagenome of CT group (received both Probio-M9 and anti-PD-1 treatments) was different from those of the two non-probiotic-receiving groups. Probio-M9 enriched sugar degradation-related pathways (such as superpathway of glycolysis and Entner–Doudoroff, pentose phosphate pathway, mannan degradation) and vitamin and amino acid synthesis pathways (such as 8-amino-7-oxononanoate biosynthesis I, biotin biosynthesis I, L-isoleucine biosynthesis IV). These functional changes improved and maintained host immunity by regulating the energy metabolism and producing beneficial metabolites, such as vitamin B7 (42–44). Modulation in the metagenomic functional potential, especially the aforementioned pathways, could be another mechanism of probiotic administration in strengthening host immunity and thus efficacy in anti-PD-1-based immunotherapy.

This study has some limitations. The mechanisms of Probio-M9 in modulating the immune system and synergizing with ICB therapy proposed in this study are mainly derived from taxonomic and functional metagenomic analyses; thus, inferences drawn here are largely observational and remain putative at this stage. Finer experiments should be designed to dissect and elucidate the physiological and molecular mechanisms of the probiotic action. Moreover, the safety of probiotic use and indeed any other novel products/management strategies is of primal importance. Although it is unlikely that probiotic intake alone would cause a drastic shift in the composition of the gut microbiome in patients, the added beneficial effects did seem to rely on the action of the probiotics in modulating patients’ gut microbiota and its function. Provided that the gut microbiota and host health are closely associated, even though the observations of this study could serve as compelling preclinical evidence supporting adjunctive use of probiotics in ICB therapy, adequate randomized controlled trials and safety assessment would still be required before such treatment could be adopted into routine clinical practice.

Nevertheless, the findings of this study supported that supplementation with probiotics after the inevitable use of antibiotics could effectively improve the outcome of and responsiveness to ICB treatment, and Probio-M9 could be considered as a candidate strain in future investigation for microbe-based synergistic tumor therapies.

## Data Availability Statement

The datasets presented in this study can be found in online repositories. The names of the repository/repositories and accession number(s) can be found below: https://www.ncbi.nlm.nih.gov/bioproject/PRJNA757579.

## Ethics Statement

This study was approved by the Special Committee on Scientific Research and Academic Ethics of Inner Mongolia Agricultural University (No. 2020-049).

## Author Contributions

HZ and ZS conceived the study. GG and TZ performed the animal experiments and prepared the samples. TM, HJ, YL, and GG carried out the metagenome analyses. GG wrote the paper with significant contributions from L-YK and TM. All authors contributed to the article and approved the submitted version.

## Funding

This research was supported by the National Natural Science Foundation of China (31720103911), China Agriculture Research System of MOF and MARA, and Science and Technology Major Projects of Inner Mongolia Autonomous Region (2021ZD0014).

## Conflict of Interest

The authors declare that the research was conducted in the absence of any commercial or financial relationships that could be construed as a potential conflict of interest.

## Publisher’s Note

All claims expressed in this article are solely those of the authors and do not necessarily represent those of their affiliated organizations, or those of the publisher, the editors and the reviewers. Any product that may be evaluated in this article, or claim that may be made by its manufacturer, is not guaranteed or endorsed by the publisher.
