# Supplementary material for: Adjunctive Probiotic Lactobacillus rhamnosus Probio-M9 Administration Enhances the Effect of Anti-PD-1 Antitumor Therapy via Restoring Antibiotic-Disrupted Gut Microbiota
Source: Front Immunol. 2021 Dec 14;12:772532. doi: 10.3389/fimmu.2021.772532 (PMC8712698; doi:10.3389/fimmu.2021.772532)
Supplement: Supplementary file 1 [file DataSheet_1.doc]

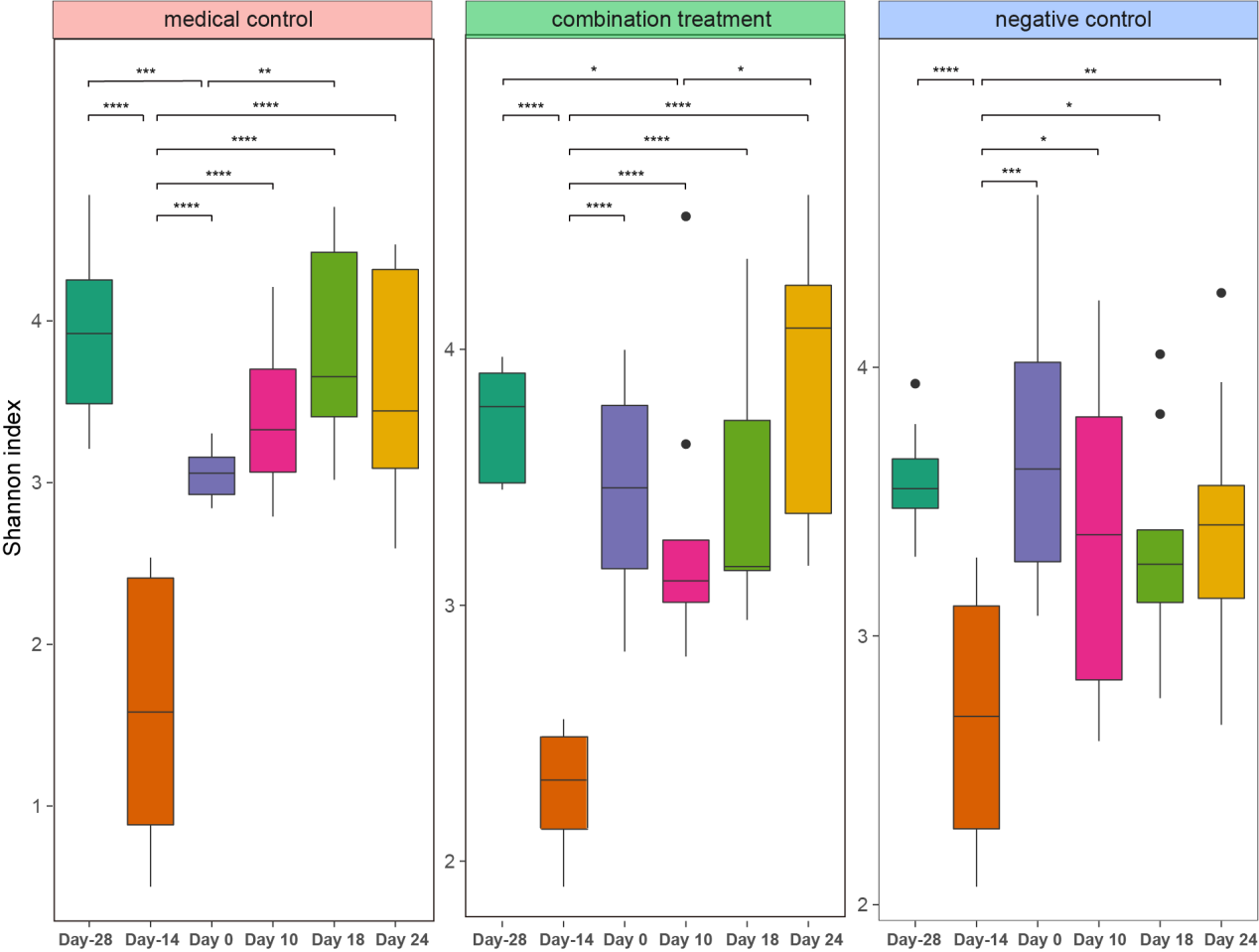


**Figure S1 Dynamic changes in alpha diversity.** (* *P*<0.05, ** *P*<0.01, *** *P*<0.001, and *P* < 0.0001)


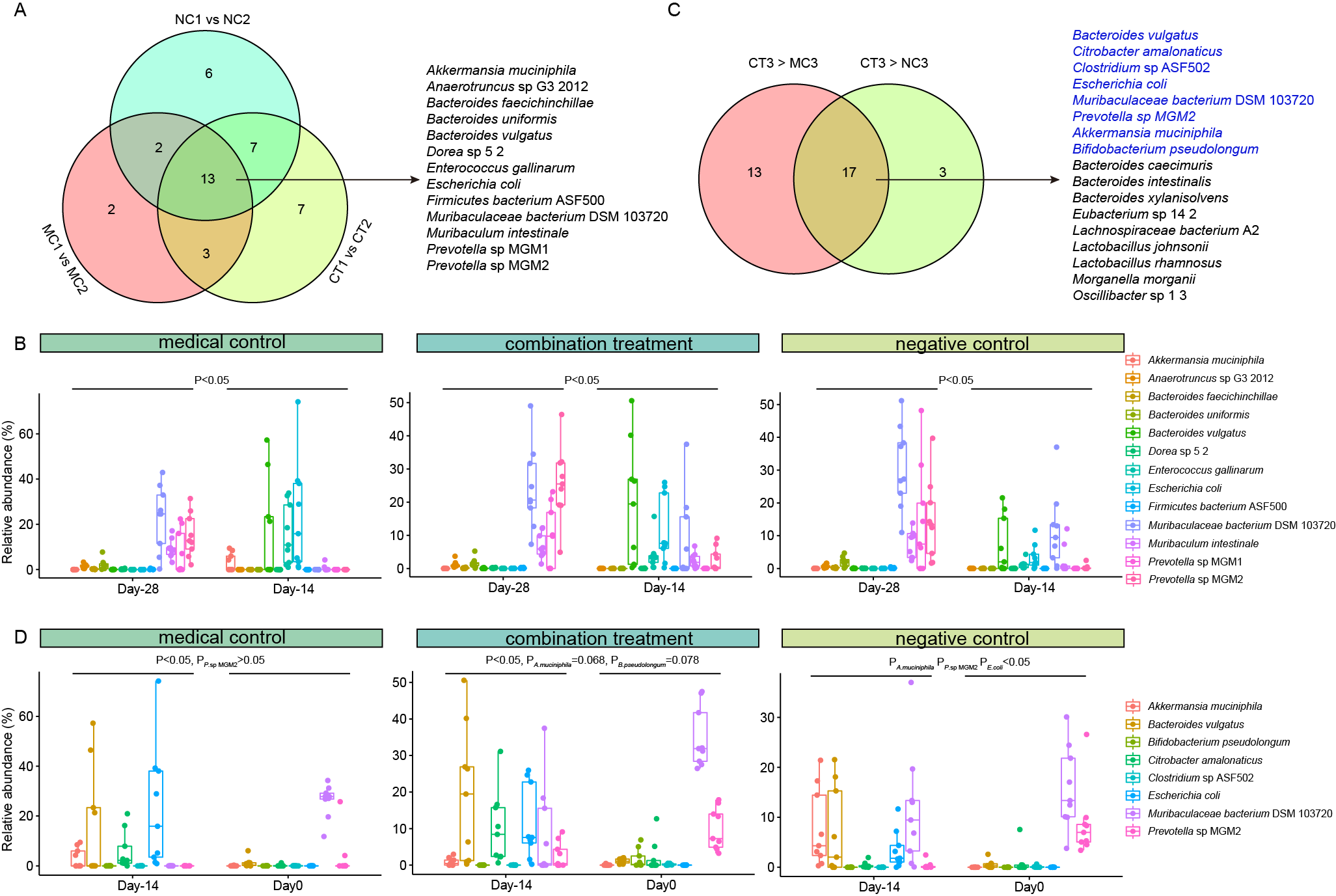


**Figure S2 Microbial species affected by administration of antibiotics and probiotics.** Venn diagrams showing (A) number of significantly changed species in each group after antibiotic treatment (*P*<0.05). (B) The relative abundance of 13 significantly changed species after antibiotic treatment, shown in (A). (C) Number of Probio-M9-modulated species in each group. Significant (*P*<0.05) or marginally significant (*P*=0.078 for *Bifidobacterium pseudolongum* and *P*=0.068 for *Akkemansia muciniphila*) differential bacterial species are written in blue. (D) The relative abundance of the eight Probio-M9-modulated species shown in (C).
